# Extraction and Sample Preparation

**DOI:** 10.1155/2015/397275

**Published:** 2015-03-24

**Authors:** Mohammad Rezaee, Faezeh Khalilian, Mohammad Reza Pourjavid, Shahram Seidi, Alberto Chisvert, Mohamed Abdel-Rehim

**Affiliations:** ^1^Nuclear Fuel Cycle Research School, Nuclear Science & Technology Research Institute, Atomic Energy Organization of Iran, P.O. Box 14395-836, Tehran, Iran; ^2^Department of Chemistry, College of Basic Science, Islamic Azad University, Yadegar-e-Imam Khomeini (RAH) Branch, P.O. Box 18155-144, Tehran, Iran; ^3^Department of Chemistry, K.N. Toosi University of Technology, P.O. Box 15875-4416, Tehran, Iran; ^4^Department of Analytical Chemistry, University of Valencia, 46100 Burjassot, Valencia, Spain; ^5^Department of Analytical Chemistry, Stockholm University, 10691 Stockholm, Sweden

As everyone knows, the role played by extraction and sample preparation in the analytical sciences cannot be overemphasized. Despite tremendous advances in chromatography, detection, and other aspects of analysis, extraction and sample preparation remain a preanalysis ritual of critical importance. It has been estimated that around 50% to 70% possibly even more of the time and effort that goes into an analytical process comprises extraction and sample preparation. Sample preparation procedure can vary in the degree of selectivity, speed, and convenience, depending on the approach and conditions used, as well as on the geometric configurations of the extraction phase and conditions. Proper design of the extraction devices and procedures facilitates rapid and convenient on-site implementation, coupled with separation/quantification and/or automation.

This special issue addresses the research studies on the sample preparation, analytical extraction, and sample clean-up techniques. For example, M. Jabłońska-Czapla reported a review on chemical speciation and provided numerous examples of the hyphenated technique usage (e.g., the LC-ICP-MS application in the speciation analysis of chromium, antimony, arsenic, or thallium in water and bottom sediment samples). T. Pérez-Palacios et al. investigated the use of a mixer mill as the homogenization tool for the extraction of free amino acids in meat samples, with the main goal of analyzing a large number of samples in the shortest time and minimizing sample amount and solvent volume. It takes less time and requires lower amount of sample and solvent than conventional techniques. N. Sher et al. studied colorimetric visible spectrophotometric quantification methods for amino acids, namely, tranexamic acid and pregabalin. Both drugs contain the amino group, and when reacted with 2,4-dinitrophenol and 2,4,6-trinitrophenol they give rise to yellow colored complexes showing absorption maximum at 418 nm and 425 nm, respectively, based on the Lewis acid base reaction.

J. A. Rodríguez et al. reported magnetic solid phase extraction of tartrazine from nonalcoholic beverages. The method involves the extraction and clean-up by activated carbon covered with magnetite dispersed in the sample, followed by the magnetic isolation and desorption of the analyte by basified methanol. The proposed methodology saves time and is less expensive than the reference method. M. Khorshid et al. reported QuEChERS (quick, easy, cheap, effective, rugged, and safe) method for extraction followed by solid phase extraction for sample purification and gas chromatography mass spectrometer, GCMS, for determination of 16 PAHs in fish at low LOQ level. E. A. Pfannkoch et al. reported combination QuEChERS and SBSE methods for extraction and concentration PAHs from fish and shellfish. I. Amin et al. reported a semiautomated extraction protocol of HCV-RNA using Favorgen RNA extraction kit. The kit provided protocol was modified by replacing manual spin steps with vacuum filtration. The assay performance was evaluated by real time qPCR based on Taqman technology. W. Wang et al. reported a high throughput sample preparation method utilizing mixed-mode solid phase extraction (SPE) in 96-well plate format for the determination of free arachidonic acid in plasma by LC-MS/MS. J. Knutsson et al. reported an uncertainty budget for the determination of fully labile Cu in water using a DGT passive sampler. H.-R. Lee et al. reported two different ionization techniques including electrospray ionization (ESI) and atmospheric pressure chemical ionization (APCI) coupled with liquid chromatography-tandem mass spectrometry (LC-MS/MS) for the analysis of cholesteryl esters (CEs). The ESI technique proved to be effective in ionizing more kinds of CEs than the APCI technique. J. Wang et al. evaluated various existing protein extraction buffers with zebrafish liver tumor samples and found that sodium deoxycholate (DOC) based extraction buffer with heat denaturation was the most effective approach for highly efficient extraction of proteins from complex tissues such as the zebrafish liver tumor. B. B. Burckhardt and S. Laeer developed sample preparation exemplified by solid phase extraction for the bioanalytical method development of low-volume assays for pediatric studies according to international agency guidelines. A. Rehman et al. suggested that* Oxalis corniculata* has good antibacterial, antifungal, and insecticidal properties and can be used for the treatment of infections and control of insects. The plant extracts could be a new source for antibiotics and pesticides with minimum noxious effects on the environment. Further studies may also lead to isolating and characterizing the active compounds of the plant extracts and elucidating their biological mechanisms of action.

